# Oesophageal pH capsule retention: case report and proposed endoscopic management

**DOI:** 10.1016/j.ijscr.2023.108917

**Published:** 2023-10-03

**Authors:** Yasir Alshareefy, Ali Alshareefy

**Affiliations:** a School of Medicine, Trinity College Dublin, The University of Dublin, Ireland; b Department of Gastroenterology, Medcare Hospital, Dubai, United Arab Emirates

**Keywords:** Oesophageal pH capsule retention, Gastroscopy, Gastroesophageal reflux disease, Endoscopy, Rare case, Education

## Abstract

**Introduction and importance:**

Gastro-oesophageal reflux disease (GORD) is a common chronic condition affecting up to 20 %. Proton pump inhibitor (PPI) is considered 1st line therapy however 10–40 % of patients do not respond adequately subsequently requiring further investigations. One of these investigations includes oesophageal pH testing via a wireless capsule placed into the oesophagus, which may remain there for up to 96 h before being self-displaced. Our report describes a rare case of oesophageal pH capsule retention and proposes a pragmatic approach to its management including endoscopic removal.

**Case presentation:**

A 33 year-old male attended our out-patient clinic with ongoing reflux symptoms and intermittent dysphagia. His response to first line therapy including lifestyle modifications and with PPIs was unsatisfactory thus a plan for an oesophageal Ph capsule study was agreed and performed. On day 4 post-procedure he reported severe dysphagia to solid foods. A Chest X-ray was performed which confirmed the presence of the capsule 7 days post-procedure. On day 12 post-procedure, gastroscopy and retrieval of the capsule was performed successfully.

**Clinical discussion:**

We recommend gastroenterologists use submucosal elevation in combination with manual traction in order to detach the capsule from the underlying mucosa, followed by retrieval using forceps to grab the thread-end of the capsule.

**Conclusion:**

We hope our report raises awareness for this rare complication as well as providing education to practicing gastroenterologists on a formal manoeuvre for successful endoscopic management of a retained oesophageal pH capsule.

## Highlights

• Capsule retention is a rare complication of oesophageal pH studies
• Suspicion should arise when patient complains of persistent dysphagia/odynophagia
• Chest X Ray should be used to confirm diagnosis of retention
• Endoscopic intervention should be used to retrieve the retained capsule
• Proposed endoscopic technique; submucosal elevation + manual traction to detach capsule followed by retrieval with forceps

## 1 Introduction

Gastro-oesophageal reflux disease (GORD) is a common chronic condition whereby gastric contents pass into the oesophagus involuntarily affecting up to 20 % [1]. Long term uncontrolled GERD can cause intestinal metaplasia whereby the squamous epithelium of the oesophagus transforms into columnar epithelium in a pre-cancerous condition known as Barrett's oesophagus which is the major cause of oesophageal adenocarcinoma and thus this highlights the importance of treating GERD adequately. Proton pump inhibitor (PPI) therapy has classically been the mainstay of therapy for GERD which works by inhibiting acid production in the stomach however 10–40 % of patients do not respond adequately to PPI therapy [2] subsequently requiring further investigations. One of these investigations includes oesophageal pH testing via a wireless capsule placed into the oesophagus, which may remain there for up to 96 h before being self-displaced, excreted and then analysed [3]. A study analysing post-marketing surveillance for the BRAVO pH capsule, which is the most commonly used device, from the FDA Manufacturer and User Facility Device Experience (MAUDE) database identified complications such as aspiration, pain, laceration, bleeding, failure to adhere to mucosa and failure of device to record or transmit data [4]. Our report describes a rare case of oesophageal pH capsule retention, up to 12 days procedure, and proposes a pragmatic approach to its management including endoscopic removal.

## 2 Methods

This case report has been reported in accordance with the SCARE criteria for case reports [5].

## 3 The case

A 33 year-old, otherwise healthy young man, attended our out-patient clinic three times over the last six months with ongoing reflux symptoms and intermittent dysphagia, with no medical background suggestive of any particular risk factors for capsule retention such as oesophageal stenosis or adhesions. His response to first line therapy including lifestyle modifications and optimal medical treatments with PPIs was unsatisfactory and he continued to seek further help. His initial gastroscopy revealed non-erosive GORD, CLO-negative gastritis and unremarkable oesophageal biopsies for eosinophilic oesophagitis.

**Fig. 1:**
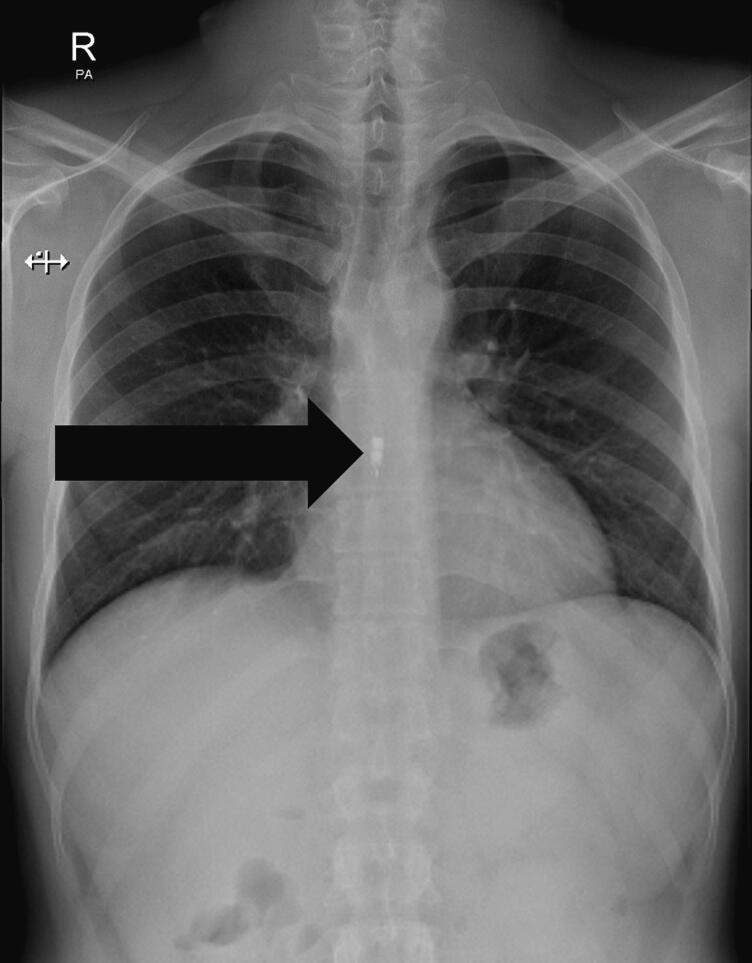
Chest X-Ray showing retention of oesophageal pH capsule with arrow pointing to retained capsule.

A plan for an oesophageal pH capsule study, off PPIs, was agreed and performed with a plan to get the receiver handed back to the endoscopy unit for analysis three days after the procedure, as per protocol. On day 4 after the procedure he reported severe dysphagia to solid foods and required analgesia, a problem which worsened over the following days necessitating a visit to the emergency department. A chest X-ray (CXR) was subsequently performed which confirmed the presence of the capsule 7 days after procedure (Fig. 1).

Technical advice from the manufacturer of the capsule was sought and patient was informed of the manufacturer's advice regarding waiting for up to 2 weeks for spontaneous detachment of the capsule. He was willing to wait and observe for few more days, however, on day 12 post-procedure the patient advised that he could no longer tolerate the dysphagia and opted for the endoscopic removal of the capsule, which was arranged the following day after a Chest X ray to confirm the presence of the retained oesophageal capsule.

**Fig. 2:**
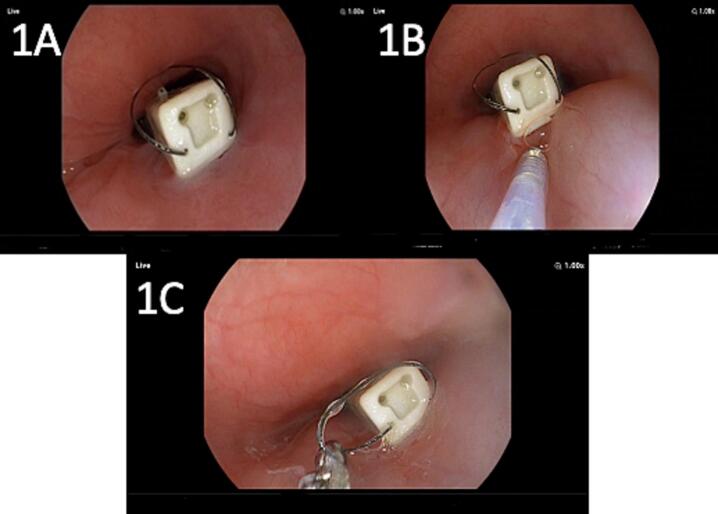
A (capsule at lower 1/3 oesophagus with surrounding granulation tissue), B (submucosal injection with saline and adrenaline), C (retrieval at thread-end via forceps).

During gastroscopy, the capsule was noted firmly attached at lower third of oesophageal wall with obvious granulation tissues at base (Fig. 2A). Submucosal elevation with saline and adrenaline was attempted (Fig. 2B) and then the capsule was pushed with a jet of water and pulled down to the stomach, held from the thread-end and retrieved (Fig. 2C). The capsule site revealed mucosal reaction but no trauma or bleeding was noted at the end of procedure.

## 4 Discussion

It is worthwhile to note that since 2009, only 30 such cases of oesophageal pH capsule retention have been noted in the literature, with the majority of reports reporting a retention duration between 9 days to 6 weeks. Of these 30 reports, 13 patients presented with chest pain, followed by 10 patients presenting with odynophagia [6].

There is a distinct lack of literature regarding risk factors for oesphageal pH capsule retention due to the limited reports of such events, thus it is difficult to predict the incidence of such a complication and risk stratify patients accordingly. One may assume that disorders resulting in oesophageal stenosis may increase the likelihood of such events such as benign structuring or hypomotility disorders, however our patient is a young, healthy male with no significant background medical history and his initial gastroscopy was unremarkable, with normal oesophageal histology on biopsy.

We recommend gastroenterologists to be more aware of the possibility of capsule retention and communicate this risk along with the other associated risks and inform the current consent form of this risk. We also recommend that clinicians have higher index of suspicion of capsule retention when, after procedure, patients continue to experience chest discomfort, dysphagia or odynophagia beyond the time window of 72 h.

The clinical approach and decision in such clinical encounters should be guided by severity of the symptoms and patient's choice which together should inform a timely and agreed clinical decision with appropriate technical support from manufacturer.

In the absence of an agreed endoscopic approach for such propose mucosal elevation with saline combined by gentle push and pull manipulation to displace the capsule off mucosa with minimal injury. An immediately before procedure, chest X ray is valuable last check of capsule retention.

Proposed Endoscopic Management of Oesophageal pH capsule retention

• 1. Submucosal injection with saline and adrenaline at the base of capsule attachment
• 2. Mechanical manipulation with a combination of push/pull manoeuvres using water or biopsy forceps to detach the capsule from the mucosal point of attachment.
• 3. Once detached, capsule site is inspected and assessed, capsule can retrieved by grasping the hanging thread ring in oesophagus if feasible or pushed down to the stomach for easier catchment.

No formal guidelines pertaining to the management of retained oesophageal pH capsules exist and such our report is the first to describe and propose a successful, practical, step-wise approach in diagnosing and endoscopically managing such a complication.

## 5 Conclusion

Our report describes a case of oesophageal pH capsule retention, occurring in a 30 year old male who presented with severe dysphagia requiring analgesia. Although a rare complication of this investigative procedure, we urge every gastroenterologist to properly inform patients of this potential risk in order for the patient to be able to make a truly informed decision regarding this procedure. We also propose a formal method of endoscopic retrieval of the retained capsule using a combination of submucosal injection to release the capsule followed by grasping the threads with forceps with subsequent removal. We hope our report becomes a source of awareness for this rare complication that may occur in the context of oesophageal pH studies, as well as providing education to practicing gastroenterologists on a formal manoeuvre for successful endoscopic management of a retained oesophageal pH capsule.

## Consent

Written informed consent was obtained from the patient for publication and any accompanying images. A copy of the written consent is available for review by the Editor-in-Chief of this journal on request.

## Funding

No funding for paper.

## Author contribution

Yasir Alshareefy; writing of paper, figure creation, submission.

Ali Alshareefy; lead clinician involved in case from diagnosis to management, writing, review of paper.

## Guarantor

Yasir Alshareefy

Ali Alshareefy

## Research registration number

N/A.

## Conflict of interest statement

No conflicts of interest.
